# Supplementary material for: Gamify4LexAmb: a gamification-based approach to address lexical ambiguity in natural language requirements
Source: PeerJ Comput Sci. 2024 Sep 19;10:e2229. doi: 10.7717/peerj-cs.2229 (PMC11419664; doi:10.7717/peerj-cs.2229)
Supplement: Supplemental Information 1 — (A) Review Checklist, (B) P1 and P2 [file peerj-cs-10-2229-s001.pdf]

APPENDIX A

No.	Checklist Elements	Assessment Y/N
User Requirements		
1.	Does framework capture user requirements from the user?	
2.	Does framework show user roles while providing requirements?	
Gamification		
3.	Is gamification exhibited in the framework?	
4.	Does selection of game elements justify according to user roles?	
5.	Does game rules properly define?	
6.	Does game rules incorporate the rewarding game elements (e.g., points)	
Ambiguity Detection and Reduction		
7.	Does POS tags suitable technique for ambiguity detection?	
8.	Is dictionary of words well-maintained?	
9.	Does ambiguity rules comply with POS tags?	
10.	Do you think user engagement during elicitation can reduce ambiguity?	
Flow and Connectivity		
11.	Do you think framework will produce the expected outcome?	
12.	Is mapping of the outcome possible on objective of the study?	
Any other Comments:		

APPENDIX B

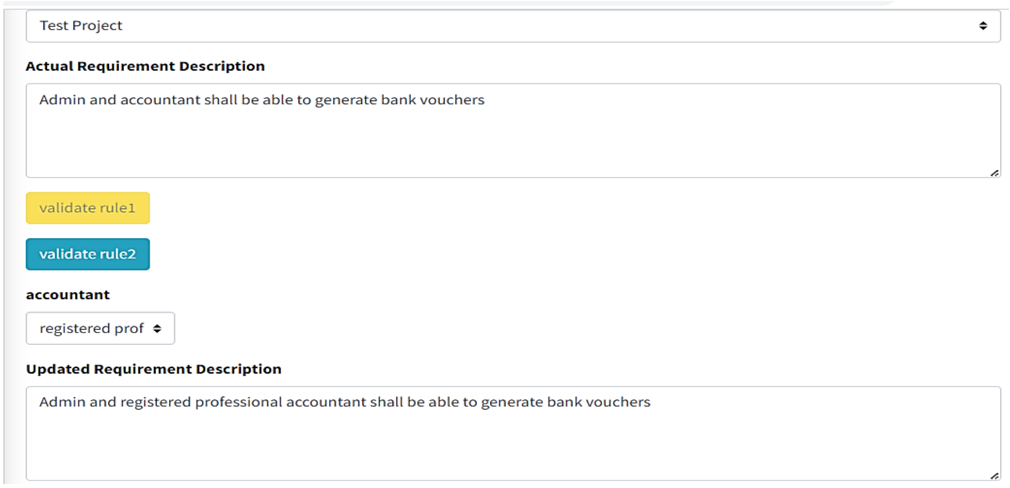

Figure: Test NL Requirement again rules P1 and P2
