# Supplementary material for: Gamify4LexAmb: a gamification-based approach to address lexical ambiguity in natural language requirements
Source: PeerJ Comput Sci. 2024 Sep 19;10:e2229. doi: 10.7717/peerj-cs.2229 (PMC11419664; doi:10.7717/peerj-cs.2229)
Supplement: Supplemental Information 3 [file peerj-cs-10-2229-s003.docx]

**Gamify4LexAmb – Prototype**

**Web Link:**

<https://crm.southload.com/gamify/login.php>

**Login Details:**

Username: [pm1@gmail.com](mailto:pm1@gmail.com)

Password: 1234
