# Supplementary material for: Gamify4LexAmb: a gamification-based approach to address lexical ambiguity in natural language requirements
Source: PeerJ Comput Sci. 2024 Sep 19;10:e2229. doi: 10.7717/peerj-cs.2229 (PMC11419664; doi:10.7717/peerj-cs.2229)
Supplement: Supplemental Information 4 [file peerj-cs-10-2229-s004.docx]

**Validation Checklist**

Reviewer 1: **Shahzad Badar**

Designation: **Software Architect**

Organization: **Enterprise Fleet Management, USA**

| **No.** | **Checklist Elements** | **Assessment Y/N** |
| --- | --- | --- |
| ***User Requirements*** | | |
| 1. | Does framework capture user requirements from the user? | Y |
| 2. | Does framework show user roles while providing requirements? | Y |
| ***Gamification*** | | |
| 3. | Is gamification exhibited in the framework? | Y |
| 4. | Does selection of game elements justify according to user roles? | Y |
| 5. | Does game rules properly define? | Y |
| 6. | Does game rules incorporate the rewarding game elements (e.g., points) | Y |
| ***Ambiguity Detection and Reduction*** | | |
| 7. | Does Rule-Based POS tags suitable technique for ambiguity detection? | Y |
| 8. | Is dictionary of words well-maintained? | Y |
| 9. | Does ambiguity rules comply with POS tags? | Y |
| 10. | Do you think user engagement during elicitation can reduce ambiguity? | Y |
| ***Flow and Connectivity*** | | |
| 11. | Do you think framework will produce the expected outcome? | Y |
| 12. | Is mapping of the outcome possible on objective of the study? | Y |
| ***Any other Comments:*** | | |
|  | | |

**Validation Checklist**

Reviewer 2: Daniyal Ahmed

Designation: System Development Leader

Organization: Cuboid AS, Norway

| **No.** | **Checklist Elements** | **Assessment Y/N** |
| --- | --- | --- |
| ***User Requirements*** | | |
| 1. | Does framework capture user requirements from the user? | Y |
| 2. | Does framework show user roles while providing requirements? | Y |
| ***Gamification*** | | |
| 3. | Is gamification exhibited in the framework? | Y |
| 4. | Does selection of game elements justify according to user roles? | Y |
| 5. | Does game rules properly defined? | Y |
| 6. | Does game rules incorporate the rewarding game elements (e.g., points) | Y |
| ***Ambiguity Detection and Reduction*** | | |
| 7. | Does Rule-Based POS tags suitable technique for ambiguity detection? | Y |
| 8. | Is dictionary of words well-maintained? | Y |
| 9. | Does ambiguity rules comply with POS tags? | Y |
| 10. | Do you think user engagement during elicitation can reduce ambiguity? | Y |
| ***Flow and Connectivity*** | | |
| 11. | Do you think framework will produce the expected outcome? | Y |
| 12. | Is mapping of the outcome possible on objective of the study? | Y |
| ***Any other Comments:*** | | |
| This idea seems to be interesting but it would be more clear how concepts are incorporated once prototyping is developed | | |
